# Thermo-Responsive Molecularly Imprinted Hydrogels for Selective Adsorption and Controlled Release of Phenol From Aqueous Solution

**DOI:** 10.3389/fchem.2018.00674

**Published:** 2019-01-24

**Authors:** Zhenhui Si, Ping Yu, Yanying Dong, Yang Lu, Zhenjiang Tan, Xiaopeng Yu, Rui Zhao, Yongsheng Yan

**Affiliations:** ^1^School of Computer Science, Jilin Normal University, Siping, China; ^2^Key Laboratory of Numerical Simulation of Jilin Province, Jilin Normal University, Siping, China; ^3^School of Chemistry and Chemical Engineering, Jiangsu University, Zhenjiang, China

**Keywords:** thermo-responsive, molecularly imprinted hydrogels, N-isopropyl acrylamide, recognition, phenol

## Abstract

In this study, thermo-responsive molecularly imprinted hydrogels (T-MIHs) were developed as an effective potential adsorbent for selectively adsorption phenol from wastewater. During the process, N-isopropyl acrylamide (NIPAm) was used as thermal responsive monomer. The obtained materials were characterized in detail by fourier transform infrared (FT-IR) spectrometer, scanning electron microscope (SEM), and thermo gravimetric analysis (TGA). A series of static adsorption studies were performed to investigate the kinetics, specific adsorption equilibrium, and selective recognition ability of phenol. Reversible adsorption and release of phenol were realized by changing temperatures. Three type of phenols, namely 3-chlorophenols (3-CP), 2,4-dichlorophenol (2,4-DCP), and 2,4,6-trichlorophenol (2,4,6-TCP) were selected as model analytes to evaluate the selective recognition performance of T-MIHs. The T-MIHs have good selectivity, temperature response, and reusability, making them ideal in applying in the controlled separation and release of phenol pollutants.

## Introduction

Molecular imprinted technique (MIT) was acknowledged as a simple and convenient method for synthesizing molecularly imprinted polymers (MIPs), which provided artificial receptor-like recognition sites for target molecule (Wang Y. X. et al., 2011; Xu et al., 2011). It generates a specific binding site by polymerizing functional monomers and template molecule in the presence of cross-linking monomers. Subsequently, template molecules are eluted from the polymers with a certain physical or chemical method, leaving a specific recognition site for template molecules in the polymer (Zayats et al., 2011). MIPs have the advantages of specific recognition, low cost, and excellent chemical, thermal, and mechanical stability (Urraca et al., 2007). Nowadays, MIPs have been applied in more and more fields, such as separation, chiral chromatographic column, solid-phase extraction, chemosensor, enzyme mimic catalysis (Li et al., 2007; Díaz-Díaz et al., 2011; Valero-Navarroa et al., 2011; Liang et al., 2012; Plewa et al., 2012; Herrero-Hernández et al., 2013), and so on.

In recent years, intelligent materials with thermal, magnetic or pH sensitivity have been introduced into the preparation of MIPs (Zhang et al., 2009; Wang et al., 2010; Fang et al., 2011; Liu et al., 2011). When thermal susceptible materials are introduced into MIPs, thermo-responsive molecularly imprinted polymers (TRMIPs) not only have high selective adsorption capacity, but can also sense temperature change and provide an appropriate response. N-isopropylacrylamide (PNIPAm) is a kind of typical thermoresponsive polymer monomer, which possesses amphiphilic properties. Above the lower critical solution temperature (LCST), which is about 32°C (Li et al., 2011), the hydrogen bond becomes weaker, forming a hydrophobic layer between molecules, resulting in a phase separation from hydrophilic state to hydrophobic state in water (Wang et al., 2015). The expansion or contraction of TRMIPs can be controlled by changing the ambient temperature. This characteristic has been utilized in applications of controlled drugs release, biological separation technology and catalysis. Wang et al. carried out a study about selective adsorption and controlled release of 2,4,5-trichlorophenol from aqueous solutions by thermo-responsive molecularly imprinted Fe_3_O_4_@carbon nanospheres (Wang et al., 2015). Xu et al. investigated the adsorption and recognizing ability of thermal-responsive molecularly imprinted polymers toward antibiotics from aqueous solutions (Xu et al., 2012).

Phenol compounds are widely used in chemicals, plastic, rubber, tanning and wood preservatives (Pan et al., 2016). The release of phenolic compounds into the aqueous environment has attracted widespread attention due to its high carcinogenicity and toxicity, as phenol leads to long-term damage to biology and the environment. According to the Environmental Protection Agency (EPA) the permissible concentration of phenol in wastewater is 1 mg/L. Regulation by the World Health Organization for limiting phenol in drinking water is 1 μg/L (Asmaly et al., 2015). Therefore, it is crucial to find efficient methods to treat phenol pollutants in water environment. In recent years, MIPs have been applied to pollutant treatment and their excellent selective adsorption capacity makes them a very promising adsorbent material. Yu et al. selective adsorption of 2,4,6-trichlorophenol from wastewater using magnetic molecularly imprinted microspheres (Yu et al., 2015a). Wang et al. carried out a study about surface molecularly imprinted polymers for highly efficient separation of 3-chlorophenol (Wang X. et al., 2011). The above-mentioned polymers represented favorably selectivity for the phenol compounds. However, to the best of our knowledge, thermo-responsive molecularly imprinted polymers for selective adsorption and controlled release of phenol from aqueous solution have been rarely reported.

In this study, it should be noted that molecular imprinting and temperature-sensitive technologies are combined. Thermo-responsive molecularly imprinted hydrogels (T-MIHs) were developed as a possible effective adsorbent for selectively adsorption phenol existent in aquatic environments. During the process, N-isopropyl acrylamide (NIPAm) was used as the thermal responsive monomer, potassium persulfate as initiator, acrylamide as monomer, N,N-methylene double acrylamide as crosslinking agent, Span-80 as surfactant, phenol as template molecule, and the obtained T-MIHs were characterized by FT-IR, SEM, and TGA. Then, T-MIHs were used as adsorbents for selective recognition phenol from wastewater. A batch mode of adsorption experiments were used to investigate the adsorption properties, such as equilibrium isotherm, kinetics, regeneration, and selectivity of T-MIHs. Moreover, the release of template molecules was realized by changing environmental temperatures.

## Experimental Section

### Materials

Cyclohexane, toluene, acrylamide (AM, 99.8%), potassium persulfate (KPS, 99%), methanol, hydrochloric acid (HCL), and ethanol were purchased from Sinopharm Chemical Reagent Co., Ltd. (Shanghai, China). Span-80, N-isopropyl acrylamide (NIPAm, 98%), and N,N'-methylene-bis-acrylamide (MBA, 99%) were purchased from Aladdin reagent Co., Ltd (Shanghai, China). 3-chlorophenols (3-CP), 2,4,6-trichlorophenol (2,4,6-TCP), 2,4-dichlorophenol (2,4-DCP) and phenol were purchased from Tianda Chemical Reagent Factory (Tianjin, China).

### Instrumentation

Infrared spectra (3,500–500 cm^−1^) were recorded on a Nicolet NEXUS-470 FT-IR apparatus (USA). A field-emission scanning electron microscope (SEM, JEOL, JSM-7001F) was used to observe the morphologies of T-MIHs. Thermogravimetric analysis (TGA) of samples was carried out using a Diamond TG/DTA instrument (STA 449C Jupiter, Netzsch, Germany) under a nitrogen atmosphere of up to 900°C with a heating rate of 5.0°C min^−1^. UV-vis adsorption spectra were measured using a UV-vis spectrophotometer (UV-2450, Shimadzu, Japan). HPLC analysis was performed on a Shimadzu LC-20A system (Shimadzu, Kyoto, Japan) equipped with a UV–vis detector. The pH of the solution was determined by PHS-2 acidimeter (The Second Analytical Instrument Factory of Shanghai, China) (Yu et al., 2015b).

### Preparation of Thermo-Responsive Molecularly Imprinted Hydrogels

The synthetic process of T-MIHs followed a literature procedure with a few modifications (Hao et al., 2012). Firstly, organic phase solutions were prepared by dissolving span-80 (0.5 g) and toluene (3.5 mL) into cyclohexane (136 mL) with vigorous stirring (360 rpm) under nitrogen at 35°C for 30 min. Then, NIPAm (3.0 g) was added to 40 mL deionized ultrapure water. After that, phenol (0.33 g), AM (1.0 g), KPS (0.4 g), and MBA (1.0 g) were successively added into the above solution. The mixture was stirred at 25°C for 30 min to form aqueous phase. Subsequently, aqueous phase was added to organic phase dropwisely during vigorous stirring (360 rpm) for 60 min. For the next step, the reaction maintained 4.0 h with vigorous stirring (360 rpm) under nitrogen at 68°C. After sufficient polymerization, the thermo-responsive molecularly imprinted hydrogels were collected and washed alternatively with deionized ultrapure water and ethanol for several times. Soxhlet extraction with a mixture of acetic acid/methanol (10:90 v/v) was used to elute template molecules from polymers, until no template molecules could be detected in the eluent. The resulting T-MIHs were finally washed several times with deionized ultrapure water and freeze-dried for 96 h. In comparison, the non-imprinted hydrogels (T-NIHs) were prepared by parallel method but with the phenol omitting.

### Procedures for Adsorption and Release of Template Phenol

The adsorption and release of phenol were studied by changing the experimental parameters (pH, temperature, initial concentration, reaction time). In adsorption isotherm studies, 10 mL of different initial concentration (from 10 to 300 mg L^−1^) of phenol solutions (pH = 6.0) were mixed with 10 mg T-MIHs or T-NIHs. The studies were carried out on a thermostatic water bath at 308.5 K until the equilibrium was established. After that, the mixture was separated and the concentration of free phenol in the supernatant was measured. The adsorption capacity (*Q*, mg g^−1^) of T-MIHs/T-NIHs was calculated according to Equation (1).

(1) $$\begin{array}{r} Q\;=\;\frac{\left(C_{0}-C\right)}{m}V \end{array}$$

Where *C* (mg L^−1^) and *C*_0_ (mg L^−1^) represent the equilibrium and initial phenol concentrations in the supernatant, respectively. V (mL) is the solution volume, m (mg) is the dry weight of T-MIHs or T-NIHs.

The imprinting factor (IF) was used to evaluate the specific property of the prepared T-MIHs, calculated from the following equation (Li et al., 2014).

(2) $$\begin{array}{r} IF=\frac{Q_{MIHs}}{Q_{NIHs}} \end{array}$$

Where *Q*_MIHs_ and *Q*_NIHs_ are adsorption capacity of T-MIHs and T-NIHs for template phenol, respectively.

The release property of phenol from the T-MIHs and T-NIHs was carried out as follows: 10 mg of the T-MIHs or T-NIHs was placed into 10 mL of phenol solutions (pH = 6.0) with an initial concentration of 100 mg L^−1^ to capture phenol at 308.5 K for 12 h and then collected from the solution by centrifugation. In order to reduce non-specific adsorption, the T-MIHs or T-NIHs were then washed with 5.0 mL of deionized ultrapure water twice. Subsequently, 10 mL of deionized ultrapure water was mixed with T-MIHs or T-NIHs, and the reaction temperatures ranged from 25 to 55°C. The release kinetics is shown in Figure S1. After 12 h of reaction, the amount of phenol released from adsorbents was determined with a UV-vis spectrophotometer.

### Procedure for Selective Binding Experiments

Ten milligram of T-MIHs or T-NIHs were mixed with 10.0 mL solution (pH = 6.0) in turn, which contained 100 mg L^−1^ of 3-CP, phenol, 2,4,6-TCP and 2,4-DCP. In comparison, the competitive adsorption of T-MIHs or T-NIHs for phenol at the presence of 100 mg L^−1^ of 2,4,6-TCP, 2,4-DCP and 3-CP were also studied. The competitive adsorption lasted 12 h in a thermostatic water bath at 308.5 K. The remnant concentration of phenol in the solution was detected using high performance liquid chromatography (HPLC). The mobile phase was consisted of deionized ultrapure water/methanol (20:80 v/v), the injection loop volume was 20 μL, and the flow rate of the mobile phase was 1.0 mL min^−1^.

## Results and Discussion

### Morphological Characterization

**Figure 1:**
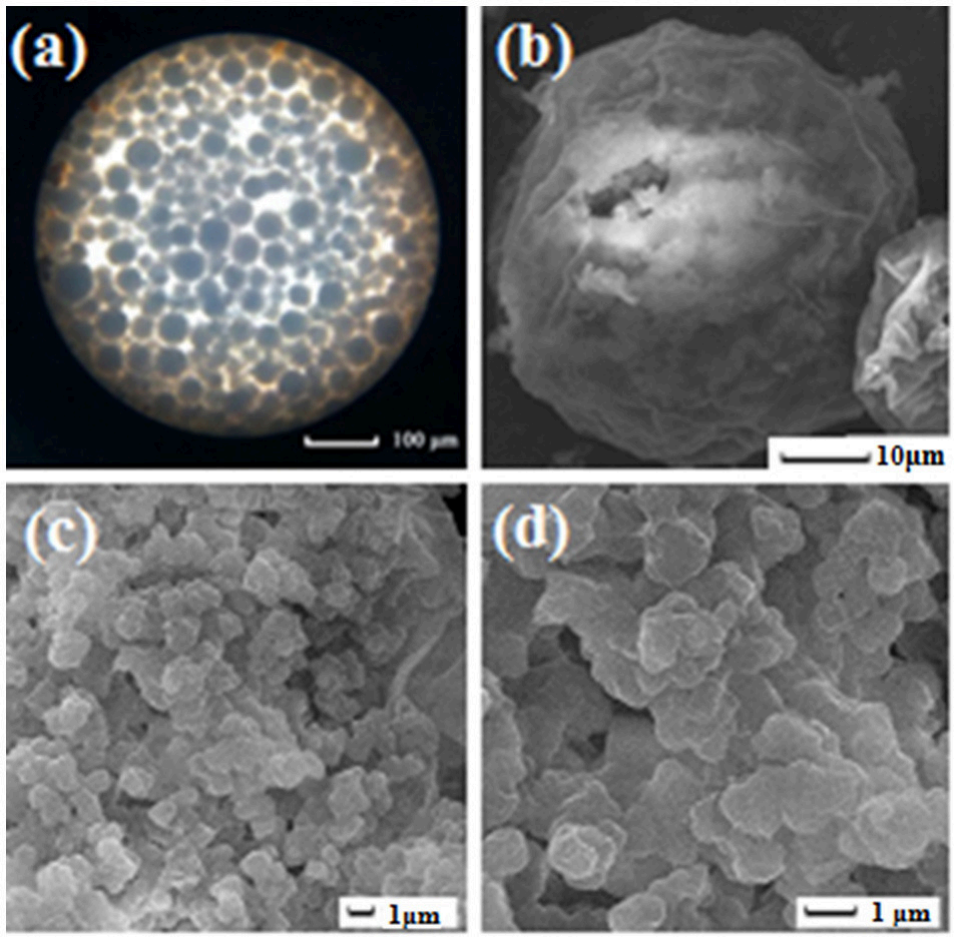
The microscope photograph **(a)** and SEM image of T-MIHs **(b–d)**.

The surface morphology of T-MIHs was observed by microscope photograph and scanning electron micrograph, respectively, which are shown in Figure 1. Through the lens of the microscope, Figure 1a showed that T-MIHs possessed spherical structure and the average size distribution of T-MIHs was about 46 ± 2 μm tested by the statistical calculation of 200 particles. From Figure 1b, it is clear that the T-MIHs particle was spherical and the surface rough, which consisted of many small-sized particles. At the high magnification in Figures 1c,d it can be seen that rough imprinted polymers with an average size of about one micron are deposited randomly on the surface of the polymer. A large number of identification sites existed between the imprinted particles, which were benefit for selective adsorption target molecular from water environment. Meanwhile, the surface morphology of T-NIHs was also observed in Figure S2, which also showed the spherical microparticles with the same size as T-MIHs, and rough surface structure.

### Infrared Spectroscopic Characterization

**Figure 2:**
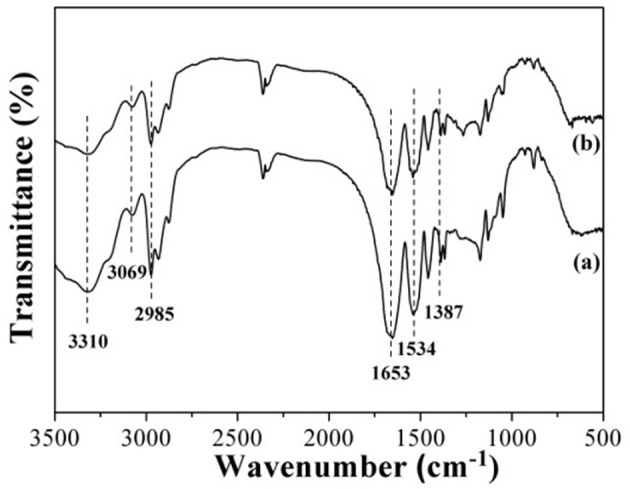
FT-IR spectra of T-MIHs **(a)** and T-NIHs **(b)**.

The FT-IR spectrometer was used to test the surface groups of the as-obtained T-MIHs and T-NIHs gel microsphere using the KBr pellet technique, which was recorded (32 scans) at room temperature between the wave number 4,000 and 400 cm^−1^. FT-IR spectra of the T-MIHs (a) and T-NIHs (b) were measured and shown in Figure 2. The contrast showed that the infrared spectra of T-MIHs and T-NIHs were similar in shape. A strong peak at 3,310 cm^−1^ could be attributed to the N-H stretching vibration of amide group (Pan et al., 2011b). Several vibration peaks were recorded at around 1,653, 1,534, and 1,387 cm^−1^, which might attributed to amide C = O stretching and deformation of methyl groups on -C(CH_3_)_2_, all characteristic of peaks of NIPAm (Tian and Yang, 2009). A strong adsorption band appeared at 2,985 cm^−1^ assigned to N-H stretching vibration of MBA. Moreover, Figure 2 exhibited a broad adsorption band during 3,100–3,000 cm^−1^, which was assigned to the C-H stretching vibration of AM (Xin et al., 2008).

### Thermo-Gravimetric Analysis

**Figure 3:**
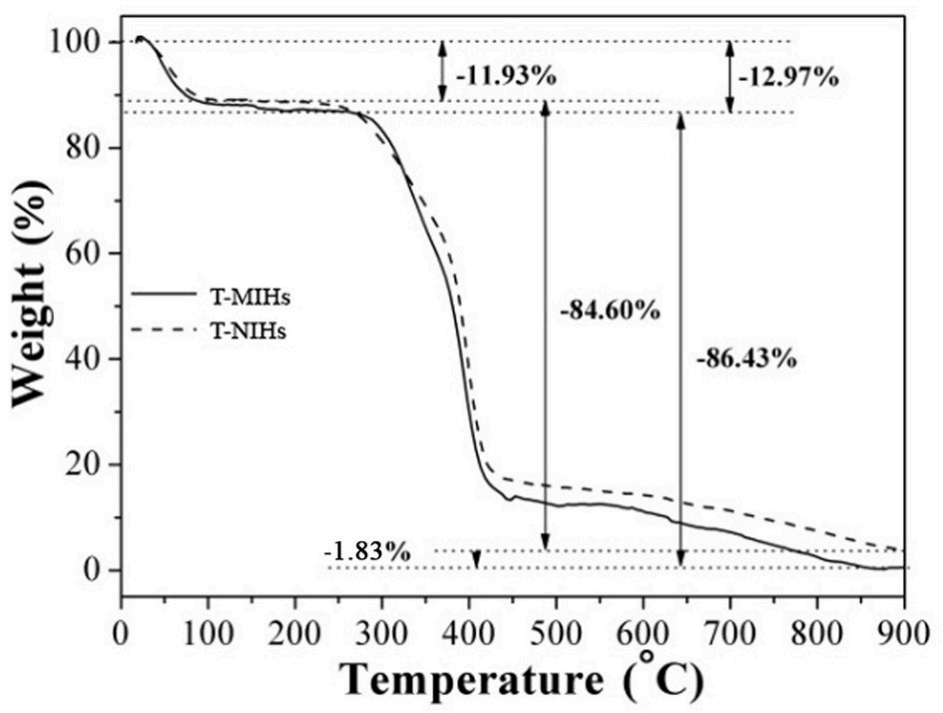
TGA curves of T-MIHS and T-NIHs.

The thermo-gravimetric analysis (TGA) curves of T-MIHs and T-NIHs are shown in Figure 3. When temperature is lower than 300°C, TGA curves showed a slight mass loss, which was due to the evaporation of residual water. Subsequently, accompanied by the temperature increase to 850°C, both curves showed significant mass loss, which was 86.43 and 84.60% for T-MIHs and T-NIHs, respectively. At this stage, the mass loss is mainly due to the decomposition of polymers. There is a difference of 1.83% in the residual quality of T-MIHs and T-NIHs, which may because of the residual template molecules in T-MIHs. When the temperature exceeds 850°C, T-MIHs and T-NIHs residues quality had no obvious change. Thus, both T-MIHs and T-NIHs exhibited good thermal stability and could meet the need for practical application in water environment.

### Thermoresponsive Property of Molecularly Imprinted Hydrogels

**Figure 4:**
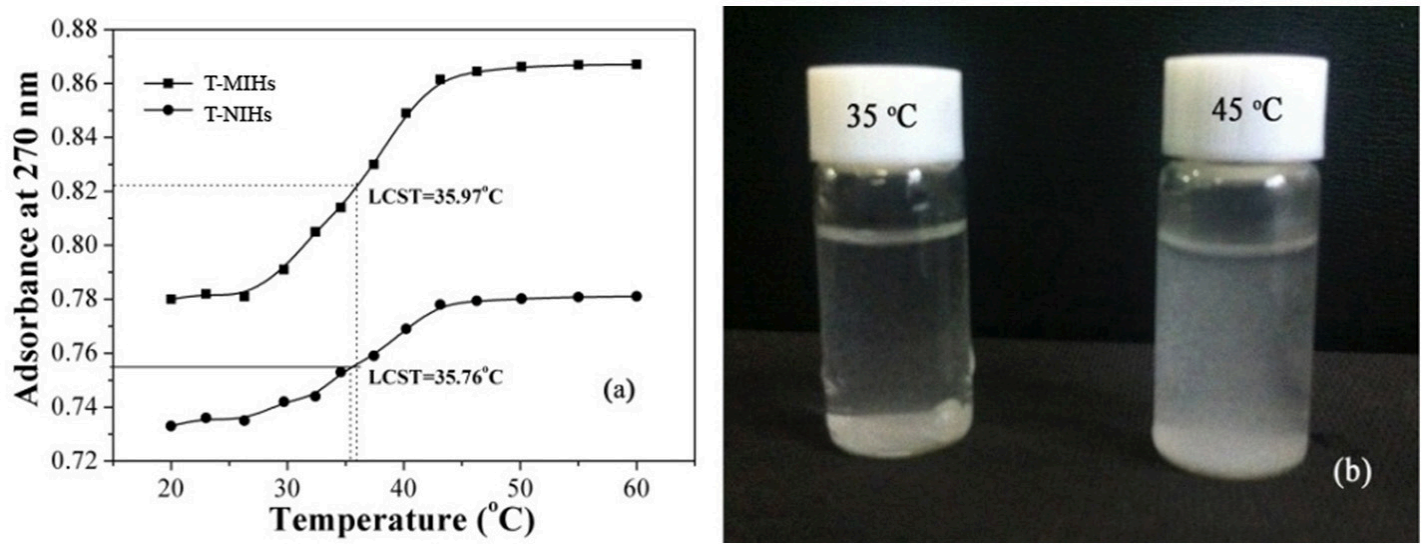
Absorbance change curve of T-MIHs and T-NIHs aqueous solution at various temperatures **(a)**, dispersion photographs under 35 and 45°C **(b)**.

In order to test the temperature responsive effect, the LCST values were determined by monitoring the absorbance change of aqueous polymer solutions (Chang et al., 2009; Pan et al., 2011a). As shown in Figure 4a, the two curves showed similar trends, the LCST values were 35.97 and 35.76°C, respectively, and the results indicated that T-MIHs and T-NIHs have remarkable temperature responsive effect. Two types of functional groups, hydrophilic and hydrophobic, existed in PNIPAm at the same time. When the temperature is lower than LCST, water molecules interact with amide groups on the molecular chain of PNIPAm to form hydrogen bonds, under the action of hydrogen bonding, PNIPAm dissolved in the water. When the temperature is higher than LCST, the hydrogen bond is weakened and the hydrophobic functional group of PNIPAm is strengthened, resulting in the two-phase separation between PNIPAm polymer and water molecule (Xu et al., 2012). Figure 4b showed that when the temperature reached 35°C, we found that hydrogels were uniformly dispersed in the solution. The shape of the template molecular was well-consistent with that of imprinted cavity and, in this condition, the best adsorption effect will be achieved. As the temperature increased, the polymer showed a certain degree of shrinkage and aggregation (Wei et al., 2008).

### Effect of pH Value on Adsorption of Phenol

**Figure 5:**
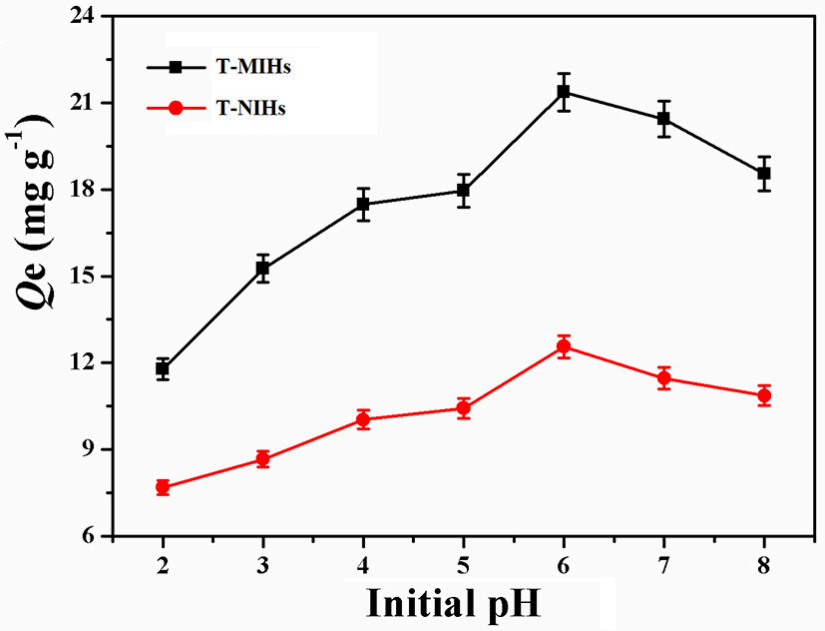
Effect of pH on adsorption of phenol.

As the key factor in the adsorption study, pH can affect the performance of adsorption. The pH of the solution affects the degree of ionization, and subsequently brings about a change in kinetics and equilibrium characteristics during the adsorption process (Yu et al., 2015b). In the experiment, 10 mg T-MIHs or T-NIHs was dispersed in 10 mL of solution which contains 100 mg L^−1^ phenol. 1.0 mol L^−1^ NH_3_·H_2_O and HCl solutions were used to adjust the initial pH value of solution, which was varied from 2.0 to 8.0. The studies were carried out on a thermostatic water bath at 25°C for 12 h. Then, the mixture was separated and the concentration of phenol in the supernatant was measured. The result was listed in Figure 5. As pH increased from 2.0 to 6.0, the adsorption capacity for T-MIHs and T-NIHs also increased significantly, and then declined in the pH range 6.0–8.0. Therefore, a pH of 6.0 was selected for further experiments.

### Thermally Modulated Adsorption of Phenol

**Figure 6:**
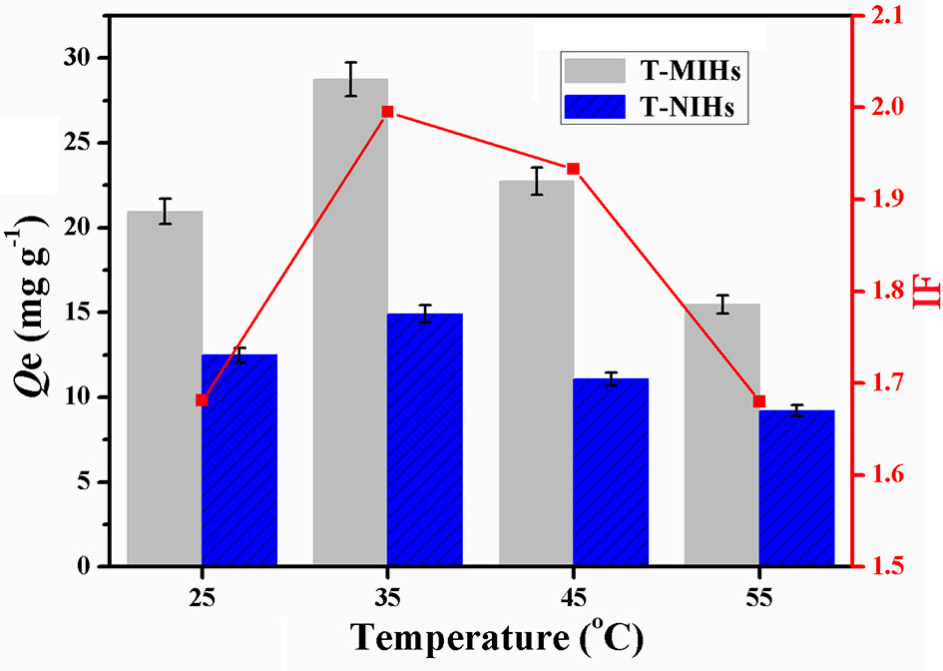
Effect of the temperature on adsorption capacity and the change curve of imprinting factor.

The thermoresponsive character was demonstrated through static adsorption experiment. In temperature impact studies, 10 mg T-MIHs or T-NIHs was dispersed in 10 mL of solution which contains 100 mg L^−1^ phenol (pH = 6.0). The reaction temperatures were controlled from 25 to 55°C. The studies were conducted in a thermostatic water bath at different temperatures for 12 h. The results were listed in Figure 6. As shown in Figure 6, the *Q* value reached the maximum value at 35°C. Such thermally modulated adsorption of phenol was closely related to thermoresponsive behavior of the imprinted hydrogels. When the temperatures reaches 35°C, as shown in Figure 4b, we found that hydrogel was uniformly dispersed in the solution. In this condition, the shape of the template phenol was well-consistent with that of the imprinted cavity, resulting in the best adsorption performance. Further increasing the temperature leads to some degrees of shrinkage and aggregation (Figure 4b), which reduced the accessibility of the active sites and further reduced the *Q*-value. For T-NIHs, the trend of the *Q*-value variation was same with the T-MIHs, but the adsorption ability was lower than that of T-MIHs. In addition, the IF value reached the maximum value at 35°C, which further demonstrated that the optimum temperature for phenol adsorption was 35°C.

### Adsorption and Release Properties of T-MIHs and T-NIHs

**Figure 7:**
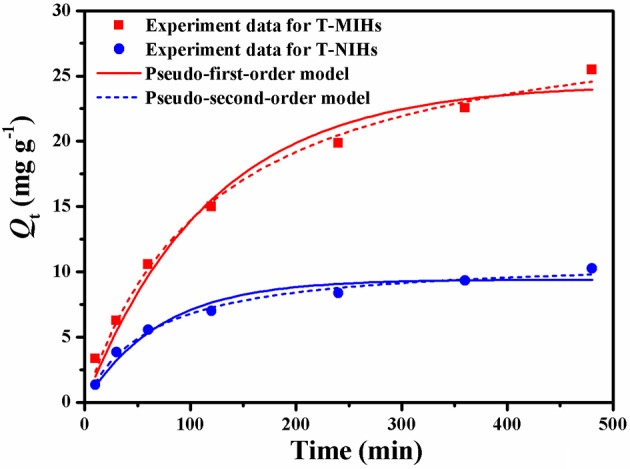
Pseudo-first-order equation and Pseudo-second-order equation for phenol adsorption onto T-MIHs and T-NIHs using non-linear regression.

At the optimum adsorption temperature, the adsorption properties of T-MIHs and T-NIHs were investigated. Adsorption kinetics showed that, compared with T-NIHs, T-MIHs absorbed more phenol because of the stronger specific adsorption (Figure 7). For both T-MIHs and T-NIHs, the Q value increased significantly in the first 60 min and then reached equilibrium within 480 min. Aiming at the examination of the adsorption mechanism such as chemical reaction and mass transfer, pseudo first-order rate equation and pseudo second-order rate equation (Supporting Information, SI) were used to analyze the kinetic data, which were obtained from experiments.

According to Equations (3–8), the values of *R*^2^, *k*_1_*, k*_2_, *h, t*_1/2_, and *Q*_*e*_ were calculated and listed in Table 1. Based on data listed in Table 1, the adsorption of T-MIHs and T-NIHs followed pseudo second-order kinetics because of the favorable fit between calculated and experimental values of *Qe* with the lower values of Akaike Information Criterion (AIC) for Pseudo-second-order kinetic model, suggesting that the former one was more reliable. Whereas, the experimental data was not well-fit for the pseudo first-order. This showed that chemical adsorption might be the rate limiting step in the adsorption process for phenol (Mazzotti, 2006).

**Table 1:** Kinetic constants for the Pseudo first-order equation and Pseudo second-order equation at 35°C.

**Samples**	**Pseudo-first-order model**				**Pseudo-second-order model**					
	***Q*_**e, c1**_**	***k*_**1**_**	***R*^**2**^**	***AIC***	***Q*_**e, c2**_**	***k*_**2**_**	***R*^**2**^**	***AIC***	***h***	***t*_**1/2**_**
	**(mg/g)**	**(L/min)**			**(mg/g)**	**(g/mg/min)**			**(mg/g/min)**	**(min)**
T-MIHs	24.39	8.42 ^*^ 10^−3^	0.977	26.83	30.73	2.70 ^*^ 10^−4^	0.992	19.08	0.27	110.7
T-NIHs	9.403	1.29 ^*^ 10^−2^	0.957	17.59	11.07	1.43 ^*^ 10^−3^	0.988	8.742	0.20	56.44

**Figure 8:**
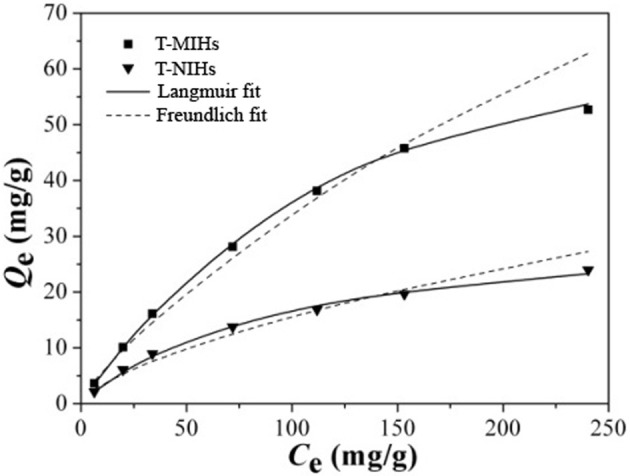
Comparison of Langmuir and Freundlich isotherm models.

A batch mode of static adsorption experiments were carried out to investigate the binding properties of phenol onto T-MIHs and T-NIHs. Adsorption equilibrium data obtained by experiments was fitted to the Langmuir and Freundlich isotherm models (SI). The two adsorption isothermal models are used to describe the equilibrium distribution of adsorbent between solution and adsorbent. Langmuir isotherm model is often used for the adsorption of monomolecular layer, while Freundlich isotherm model is often used for the adsorption of multi-molecular layer (Li et al., 2012a). The applicability of isothermal models to adsorption behavior is expressed by the correlation coefficient (*R*^2^). Table 2 lists the adsorption isotherm constants for T-MIHs and T-NIHs at optimum reaction temperature. Moreover, the regression curves of Langmuir and Freundlich isotherm models for phenol adsorption onto T-MIHs and T-NIHs are illustrated in Figure 8.

**Table 2:** Adsorption isotherm constants at 35°C.

**Isotherm models**		**T-MIHs**	**T-NIHs**
Langmuir equation	*R^2^*	0.993	0.991
	*Q*_m.c_ (mg/g)	77.09	32.89
	*K*_L_ (L/mg)	0.006	0.009
	*R*_L_	0.38	0.28
Freundlich equation	*R*^2^	0.984	0.977
	*K*_F_ ((mg g^−1^)(1 mg^−1^)^1/n^)	0.97	0.66
	*n*	1.30	1.51

As it can be seen from the adsorption isotherms in Figure 8, with the concentration of phenol increasing from 10 to 240 mg mL^−1^, the *Q*-value for T-MIHs and T-NIHs increased significantly. Moreover, the *Q*-values of T-NIHs were all lower than those of T-MIHs due to the imprinted effect. The adsorption isotherm constants for T-MIHs and T-NIHs are shown in Table 2. Obviously, compared with Freundlich isotherm model, the correlation coefficient was more suitable for the Langmuir isotherm model. Moreover, T-MIHs/T-NIHs had good applicability for the Langmuir isotherm model, indicating monolayer molecular adsorption for T-MIHs/T-NIHs (Lin et al., 2014).

**Figure 9:**
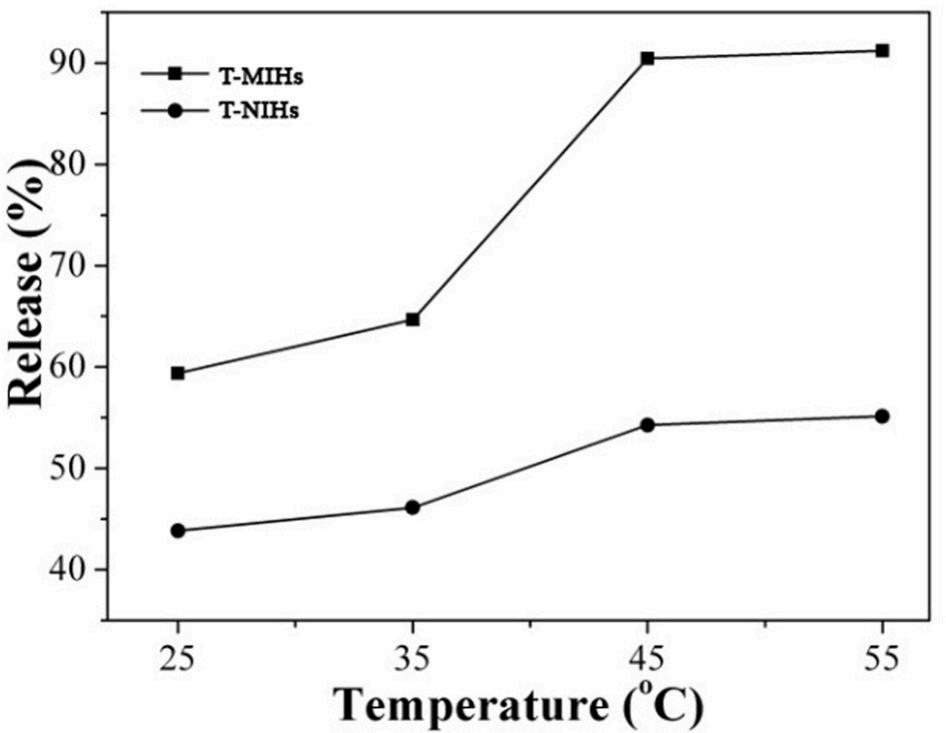
Effect of temperature on the release rate of phenol.

**Figure 10:**
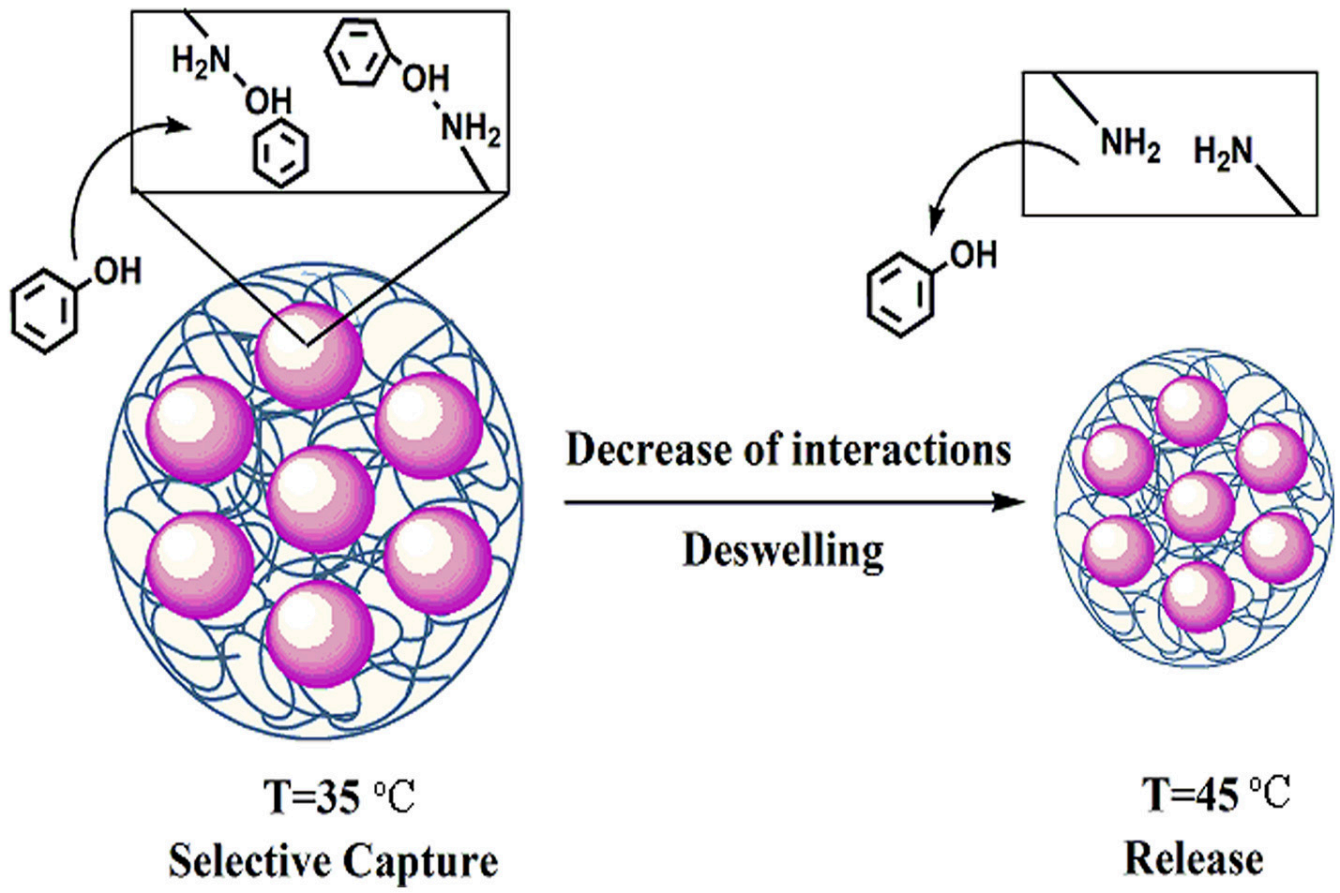
Schematic illustration of possible mechanism for temperature effect on phenol release.

T-MIHs and T-NIHs with captured phenol were applied in subsequent release experiments. Figure 9 shows the released amounts of phenol after its release at different temperatures for 12 h. It can be seen that the release percentage of phenol gradually increased with the increasing temperature. For example, considering T-MIHs, the release rates of phenol at four temperatures are 59.37, 64.67, 90.45, and 91.21%, respectively. When the temperature exceeded 45°C, the release rate of absorbed phenol could reach 90%. The reason for this phenomenon could be that increased temperature made the T-MIHs/T-NIHS more hydrophobic and caused deswelling, resulting in the interactions between the hydrogels and phenol weakened, and the sites were too small to capture the template molecular, triggering the release of phenol, as shown in Figure 10. The results showed that the temperature sensitive property could be effectively applied in the treatment of pollutants, in which the target molecular could effectively switch adsorption and release by changing temperatures.

### Reusability

**Figure 11:**
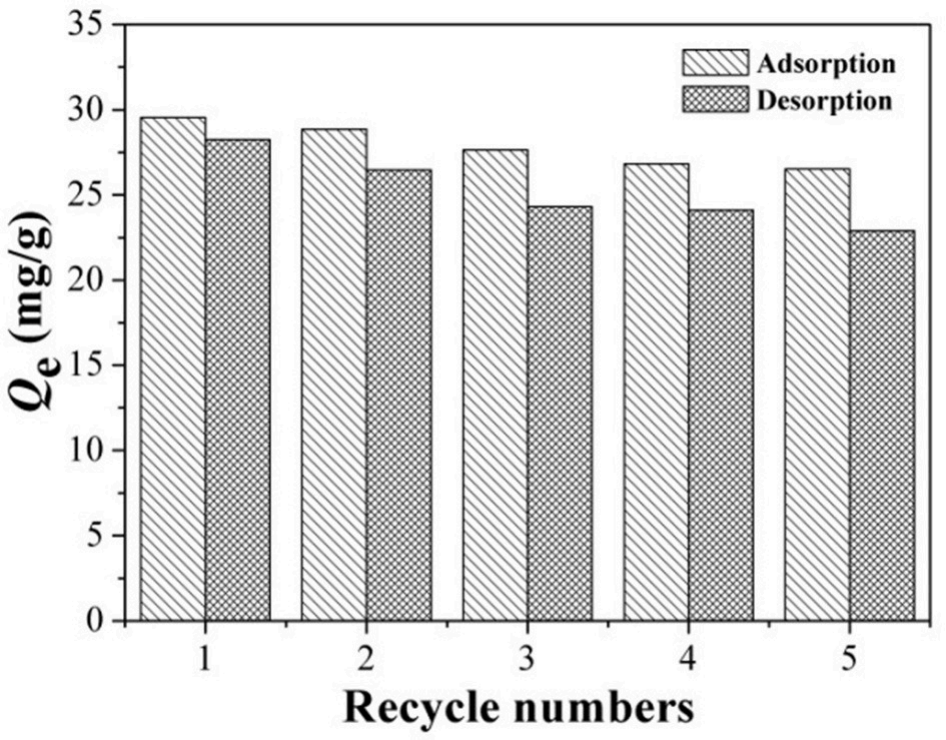
Five recycles of regeneration at optimum condition.

In order to verify the recycle and stability of the T-MIHs, five regeneration cycles were carried out under the same experiment conditions. Firstly, isothermal adsorption at 308 K was performed on T-MIHs. Subsequently, the mixture of acetic acid and methanol was used to elute the adsorbed phenol at 318 K. Ultimately, deionized ultrapure water washed the T-MIHs to neutral conditions for the next adsorption-desorption cycle. Figure 11 shows the final results of five cycles of adsorption. After five regenerations, the loss of adsorption percentage for T-MIHs toward target molecular was around 6.78%, and the TGA curve of cycled sample was matched with that of fresh T-MIHs (as shown in Figure S3), demonstrating remarkable chemical and structure stability of T-MIHs.

### Selectivity Analysis of the T-MIHs

**Figure 12:**
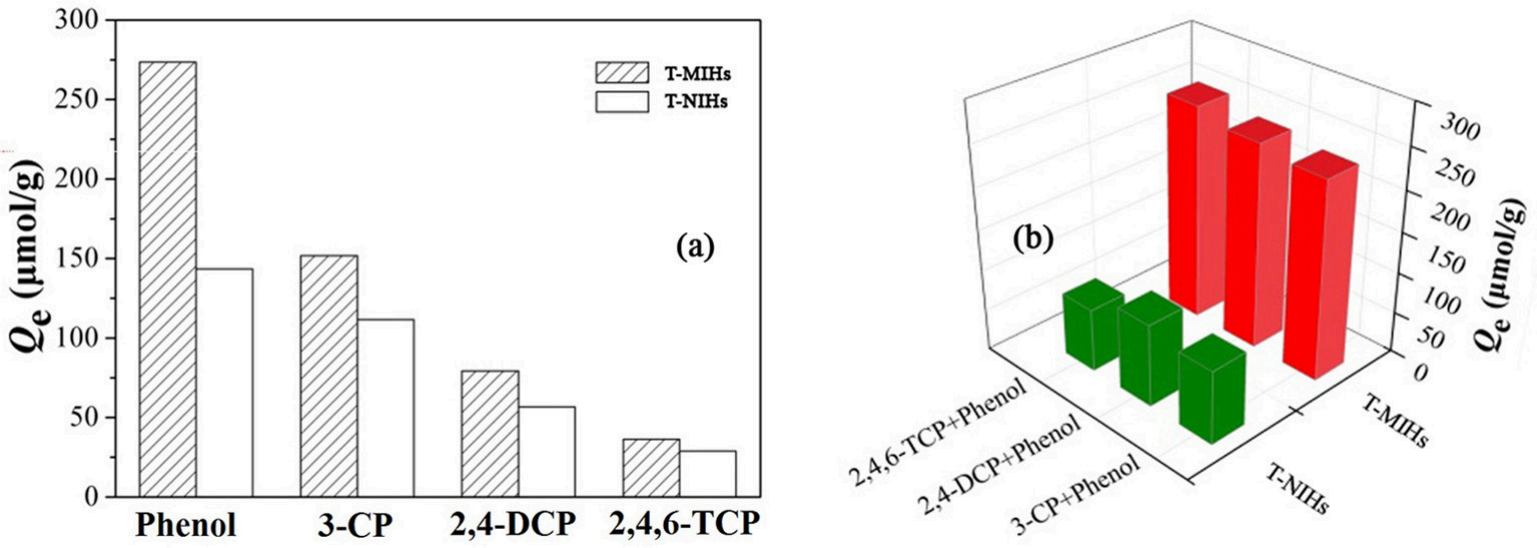
Adsorption selectivity of phenol onto T-MIHS and T-NIHs in single **(a)** and dual **(b)** solute.

To measure the selectivity of T-MIHs, the recognition of phenol was compared with 2,4,6-TCP, 2,4-DCP and 3-CP, respectively. The molar mass and molecular structures of phenolic compounds are shown in Table 3. A series of static adsorption experiments were carried out in single and binary aqueous solutions, respectively. The initial solution concentration was 100 mg L^−1^ and pH value was 6.0. Adsorption capacity of T-MIHs and T-NIHs for phenol in single and binary aqueous solutions is shown in Figure 12. As is shown in Figure 12, T-MIHs possessed the best adsorption capacity to target molecular among the four adsorbents. The results further proved that T-MIHs possessed specific recognition for phenol. In binary aqueous solutions, due to the influence of competitive compounds, the adsorption capacity of T-MIHs and T-NIHs decreased slightly and, even so, T-MIHs still showed the specific adsorption for the target phenol. The two results above indicated that the selectivity of T-MIHs for phenol was still obvious. Here, acrylamide, N-isopropyl acrylamide and N,N-methylene double acrylamide were used as functional monomer, thermo-responsive monomer and cross-linker, respectively, to prepare imprinted polymers. Thus, the hydrogen-bonding interaction can be formed between phenolic hydroxyl groups of template molecule phenol and amino- (amide) groups from imprinted sites, the energy of which is between 25 and 40 kJ mol^−1^. Phenol, 2,4,6-TCP, 2,4-DCP and 3-CP are different in structure, size and functional groups, resulting in the different conformation memory of specific binding sites (Pan et al., 2013). Also, the size, sharpness and structure of imprinted sites provided a strong interaction.

**Table 3:** Molar mass and molecular structures of the phenolic compounds.

**Phenolic compounds**	**Molar mass (g/mol)**	**Structure**
Phenol (template molecule)	94.11	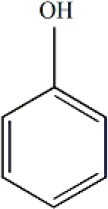
2,4-DCP (competition content)	163	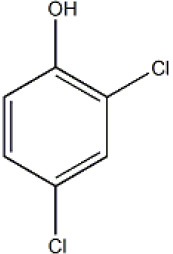
3-CP (competition content)	128.56	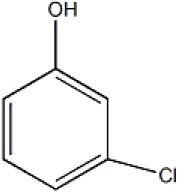
2,4,6-TCP (competition content)	197.44	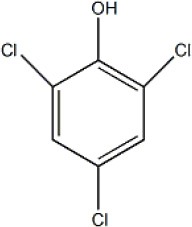

## Conclusion

In this study, combining an imprinted technique and temperature response, molecularly imprinted hydrogels were successfully synthesized by suspension polymerization. A series of adsorption experiments indicated that T-MIHs performed well, for instance in selective recognition, excellent adsorption capacity and fast adsorption kinetics, and the target molecular could effectively switch adsorption and release by changing temperatures. We believe that the smart imprinting systems should be promoted at the forefront of MIHs, which possessed the stimulus-responsive recognition. This kind of material has great application potential for drug release, separation, protein recognition and so on.

## Author Contributions

ZS: the complementary adsorption experiments and article revision. PY: study of adsorption mechanism. YD: material preparation. YL: kinetic analysis. ZT: thermodynamic analysis. XY and RZ: characterization. YY: adsorption experiment.

### Conflict of Interest Statement

The authors declare that the research was conducted in the absence of any commercial or financial relationships that could be construed as a potential conflict of interest.
