# Supplementary material for: Thermo-Responsive Molecularly Imprinted Hydrogels for Selective Adsorption and Controlled Release of Phenol From Aqueous Solution
Source: Front Chem. 2019 Jan 24;6:674. doi: 10.3389/fchem.2018.00674 (PMC6357936; doi:10.3389/fchem.2018.00674)
Supplement: Supplementary file 1 [file Data_Sheet_1.pdf]

Supporting Information

1. Equations for describing equilibrium performances.

The pseudo first-order equation can be expressed as linear and non-linear forms by equations (3) and (4), respectively (Yu et al., 2015c):

$$ln(Q_e - Q_t) = lnQ_e - k_1 t \quad (3)$$

$$Q_t = Q_e - Q_e e^{(-k_1 t)} \quad (4)$$

The pseudo second-order equation can be expressed as linear and non-linear forms by equations (5) and (6), respectively (Yu et al., 2015b):

$$\frac{t}{Q_t} = \frac{1}{k_2 Q_e^{2}} + \frac{t}{Q_e} \quad (5)$$

$$Q_t = \frac{k_2 Q_e^{2} t}{1 + k_2 Q_e t} \quad (6)$$

Where Q_t (mg g^{-1}) is the amount adsorbed at various time t, while Q_e (mg g^{-1}) is the amount adsorbed at equilibrium time. k_1 (min^{-1}) is the equilibrium rate constant of the pseudo-first-order model, the value of k_1 can be obtained by plotting $ln(Qe - Qt)$ versus t. k_2 (g mg^{-1} min^{-1}) is the pseudo-second-order model rate constant calculated from the plot of t/Q_t versus t.

Based on the pseudo second-order kinetic model, the initial adsorption rate (h, mg g^{-1} min^{-1}), and half-adsorption time ($t_{1/2}$, min) were calculated according to the following equations (7) and (8) (Yu et al., 2015b):

$$h = k_2 Q_e^2 \quad (7)$$

$$t_{1/2} = \frac{1}{k_2 Q_e} \quad (8)$$

The linear and non-linear forms of the Langmuir isotherm models are expressed by the following equations (9) and (10), respectively (Yu et al., 2015c):

$$\frac{C_e}{Q_e} = \frac{1}{Q_m K_L} + \frac{C_e}{Q_m} \quad (9)$$

$$Q_e = \frac{K_L Q_m C_e}{1 + K_L C_e} \quad (10)$$

Where C_e is the equilibrium concentration of adsorbate (mg L^{-1}), Q_e is the equilibrium adsorption capacity (mg g^{-1}), Q_m (mg g^{-1}) is the maximum adsorption capacity, K_L (L

mg^{-1}) represents the Langmuir adsorption constant.

For predicting the favorability of an adsorption system, the Langmuir equation can also be expressed in terms of a dimensionless separation factor R_L (Yu et al., 2015b).

$$R_L = \frac{1}{1 + C_m K_L} \qquad (11)$$

Where C_m (mg L^{-1}) is the maximal initial concentration of solutions. The value of R_L indicates whether the type of the isotherm is favorable ($0 < R_L < 1$), unfavorable ($R_L > 1$), linear ($R_L = 1$), or irreversible ($R_L = 0$).

The linear and non-linear forms of the Freundlich isotherm models are expressed by the following equations(12) and (13), respectively (Yu et al., 2015b) :

$$lnQ_e = lnK_F + \frac{1}{n} lnC_e \qquad (12)$$

$$Q_e = K_F C_e^{1/n} \qquad (13)$$

Where K_F ((mg g^{-1})(mg^{-1})$^{1/n}$) is the Freundlich adsorption constant and $1/n$ is the heterogeneity factor measuring the adsorption intensity or surface heterogeneity. The value of $1/n$ ranging from 0.0 to 1.0 represents a normal Langmuir adsorption condition; otherwise, it was indicative of cooperative adsorption (Li et al., 2012b).

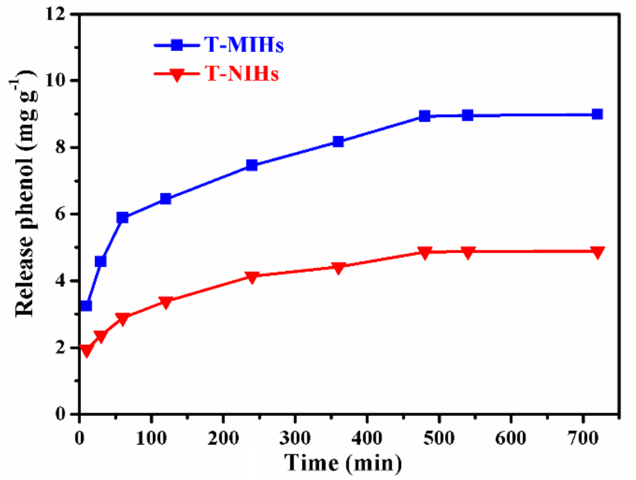

Fig. S1 Release kinetics of T-MIHs and T-NIHs at 45 °C.

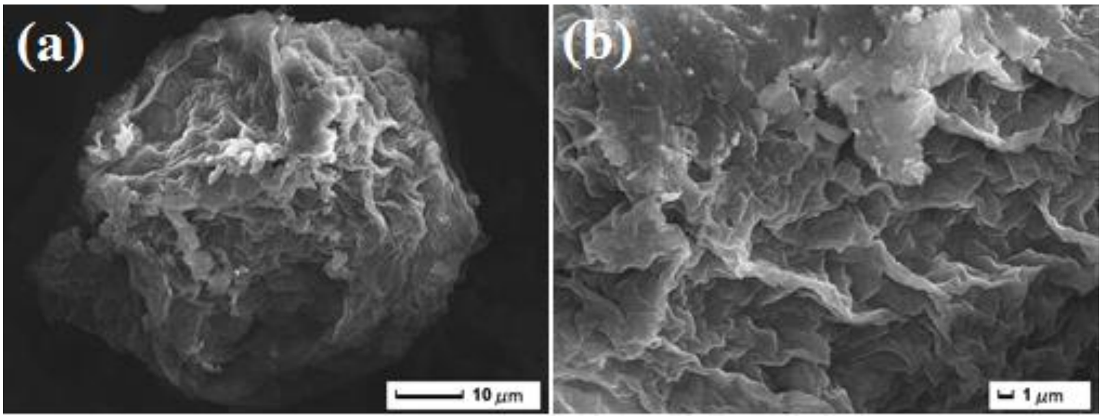

Fig. S2 SEM images of T-NIHs.

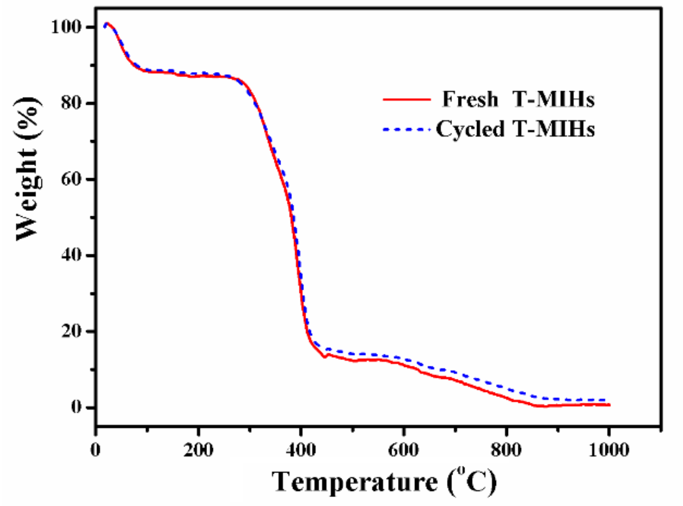

Fig. S3 The TGA curves of fresh T-MIHs and cycled T-MIHs
